# Supplementary material for: Mapping the conformational energy landscape of Abl kinase using ClyA nanopore tweezers
Source: Nat Commun. 2022 Jun 20;13:3541. doi: 10.1038/s41467-022-31215-5 (PMC9209526; doi:10.1038/s41467-022-31215-5)
Supplement: Supplementary file 1 — Supplementary Information [file 41467_2022_31215_MOESM1_ESM.pdf]

# Supplementary Information: Mapping the conformational energy landscape of Abl kinase using ClyA nanopore tweezers

M. Chen et al.

## Contents

Supplementary Fig. 1. Schematics of open pore current ($I_O$) and blocked pore current ($I_B$). ....... 3
Supplementary Fig. 2. Michaelis-Menten kinetics analysis of Abl kinases. ................................ 4
Supplementary Fig. 3. Trapping time of Abl kinases at different applied voltages. .................... 5
Supplementary Fig. 4. Representative traces of Abl trapped in a ClyA-AS nanopore at different voltages. ........................................................................................................................ 6
Supplementary Fig. 5. Examples of partial S1, S2 and complete S1, S2 events. ...................... 7
Supplementary Fig. 6. A representative trace of $Abl_{C4pos}$ trapped in a ClyA-AS nanopore. ......... 8
Supplementary Fig. 7. Zoom in trace of S1 and S2 states from apo Abl trapping signal. ............ 9
Supplementary Fig. 8. Representative traces of ligand interactions with $_{N4pos}$Abl. ......................10
Supplementary Fig. 9. Effect of $MgCl_2$ on ATP binding with $_{N4pos}$Abl. ........................................11
Supplementary Fig. 10. Representative traces and corresponding histograms of S2 state during ligand interactions with $_{N4pos}$Abl. ..........................................................................................12
Supplementary Fig. 11. Comparison of $_{N4pos}$Abl binding with imatinib and $_{N4pos}$Abl binding with ATPγS and Abltide. ...........................................................................................................13
Supplementary Fig. 12. Interaction of ATPγS with $_{N4pos}$G321V. ..............................................14
Supplementary Fig. 13. SDS-PAGE analysis of ClyA-AS and Abl kinases. ..............................15
Supplementary Table 1. Michaelis-Menten kinetics analysis of Abl kinases. ............................16
Supplementary Table 2. Voltage dependent trapping time analysis of Abl kinases. .................17
Supplementary Table 3. Characteristics of apo $_{N4pos}$Abl and $_{N4pos}$G321V current signals. ..........18
Supplementary Table 4. Occupancy of S1 sub-states of $_{N4pos}$Abl under different conditions. .....19
Supplementary Table 5. Trapping time of different Abl constructs and their S1, S2 dwell times at -80 mV. ......................................................................................................................................20
Supplementary Table 6. Kinetic and thermodynamic parameters for apo $_{N4pos}$Abl. .....................21
Supplementary Table 7. Primers for Abl variants cloning. .......................................................22

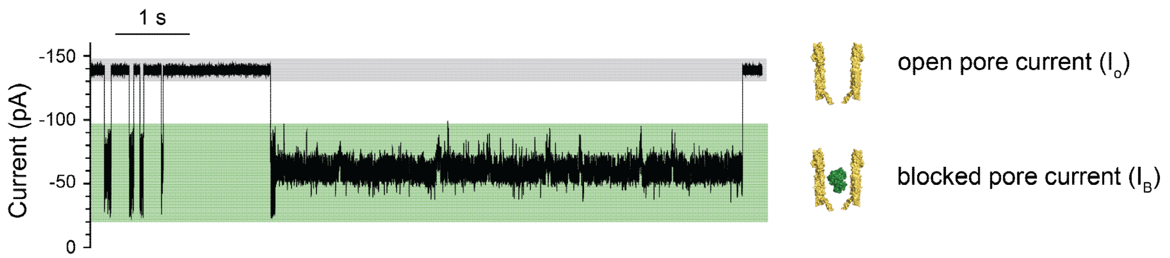


**Supplementary Fig. 1.** Schematics of open pore current ($I_O$) and blocked pore current ($I_B$). Open pore current ($I_O$, highlighted in grey) was detected when no target analytes interacted with the nanopore, blocked pore current ($I_B$, highlighted in green) was detected when an analyte molecule (here is the Abl kinase) entered the nanopore. Of note the blocked pore current may contain different levels depends on the analytes. Models for open pore and blocked pore were shown next to the current trace with ClyA-AS and Abl kinase colored in yellow and green, respectively. The current traces were collected at −80 mV in 150 mM NaCl, 100 mM Tris-HCl pH 7.5 buffer, with ~100 nM Abl kinase added to *cis* chamber, at 22 ºC.

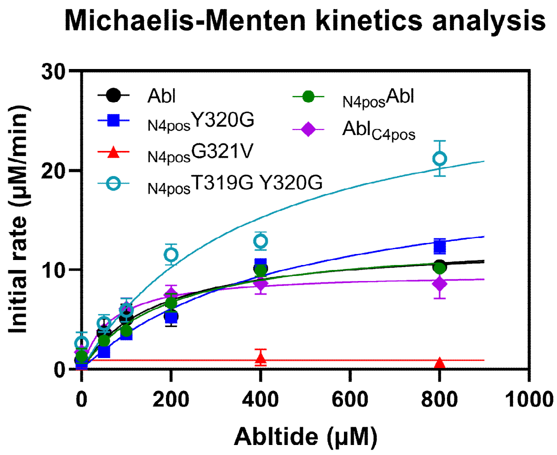


**Supplementary Fig. 2.** Michaelis-Menten kinetics analysis of Abl kinases (Abl, $Abl_{C4pos}$, $_{N4pos}Abl$, $_{N4pos}G321V$, $_{N4pos}Y320G$, and $_{N4pos}T319G$ Y320G). Data were represented as mean ± SD, n=3 independent replicates. Source data are provided as a Source Data file. N4pos or C4pos indicate Abl constructs with a positively charged peptide tail at its N-terminus, termed $_{N4pos}Abl$, or to its C-terminus, named $Abl_{C4pos}$ (peptide sequence for N4pos tag; C4pos tag: GGSKKRK).

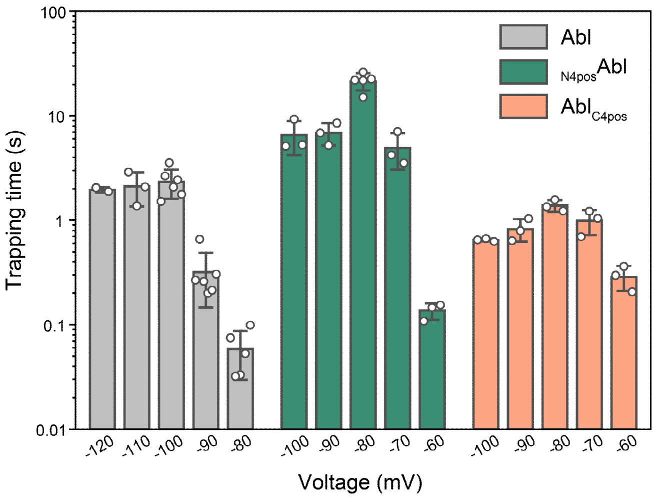


**Supplementary Fig. 3.** Trapping time of Abl kinases (Abl, $Abl_{C4pos}$ and $_{N4pos}Abl$) at different applied voltages. Note that the y axis is logarithmic. Data were represented as mean ± SD, n=6 independent replicates for Abl at -90 mV and -100 mV; n=5 independent replicates for Abl at -80 mV and $_{N4pos}Abl$ at -80 mV; n=3 independent replicates for Abl at -110 mV, $_{N4pos}Abl$ at -60, -70, -90, -100 mV, and $Abl_{C4pos}$ at -60~-100 mV; n=2 independent replicates for Abl at -120 mV. Source data are provided as a Source Data file.

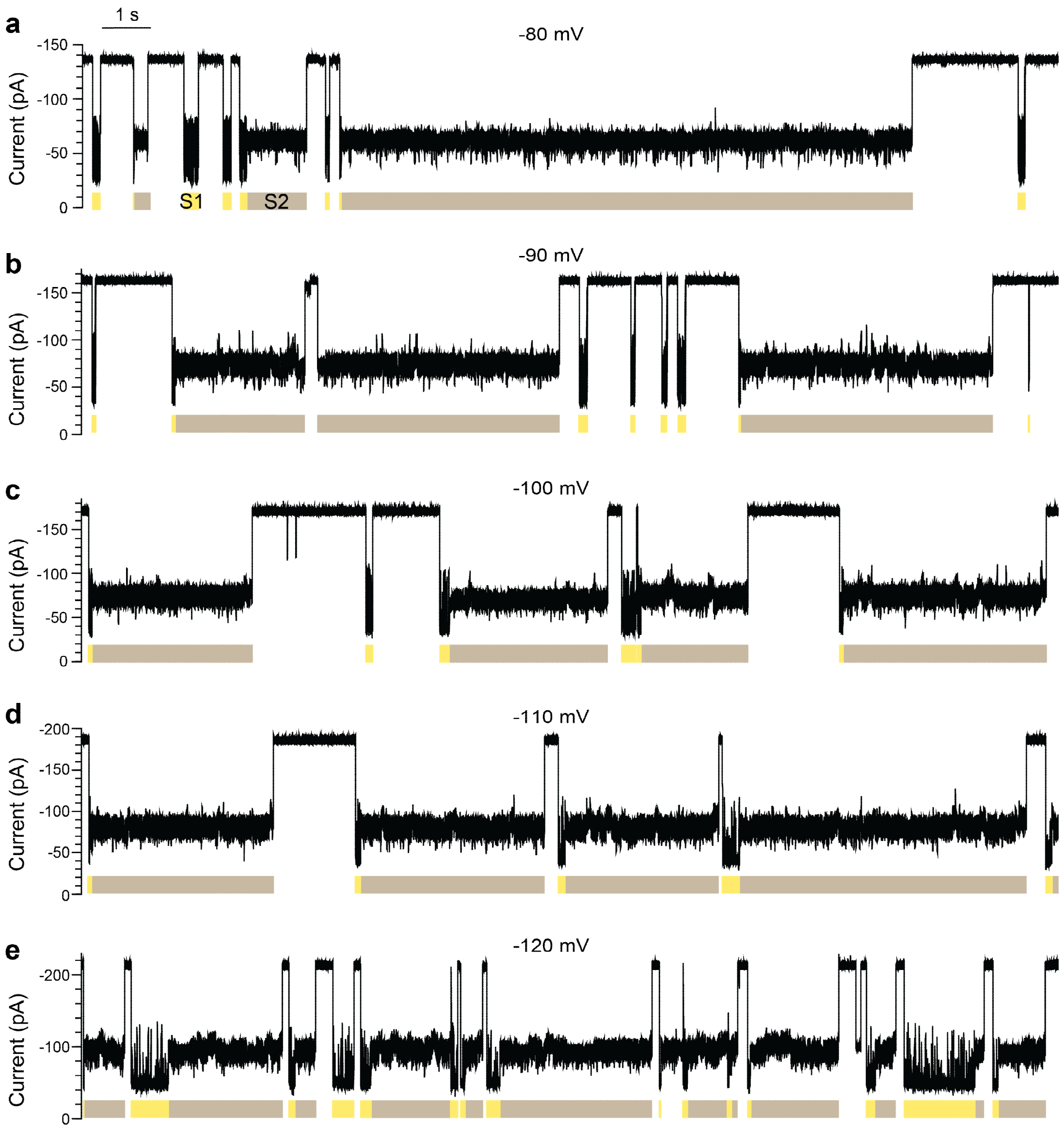


**Supplementary Fig. 4.** Representative traces of Abl trapped in a ClyA-AS nanopore at different voltages (a-e). Signal pattern S1 (yellow) and S2 (brown) are observed at all tested voltages. The current traces were collected in 150 mM NaCl, 100 mM Tris-HCl, pH 7.5, with ~100 nM Abl kinase added to *cis* chamber, at 22 ºC.

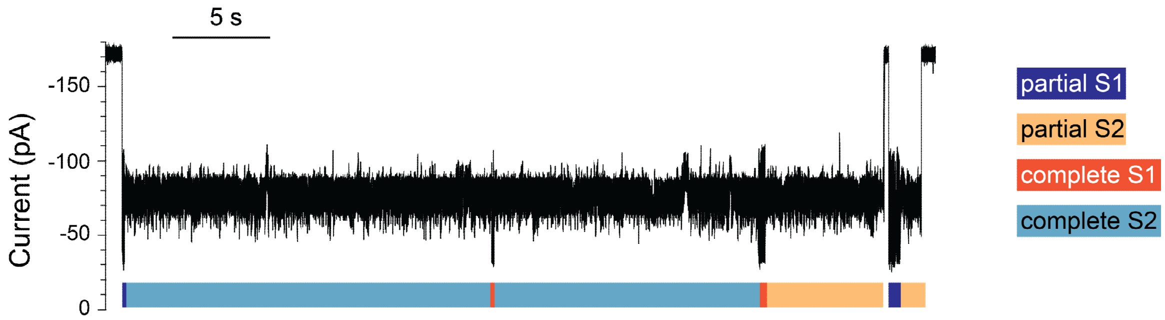


**Supplementary Fig. 5.** Examples of partial S1 (dark blue), partial S2 (apricot), complete S1 (orange) and complete S2 (light blue) events. The S1 or S2 events at the beginning and those at the end of a trapping event are considered as partial events and were discarded while calculating $\tau_{S1}$ and $\tau_{S2}$, as their true dwell times were interrupted by Abl entering and exiting the ClyA-AS nanopore. The current traces were collected at −100 mV in 150 mM NaCl, 100 mM Tris-HCl, pH 7.5, with ~100 nM Abl added to *cis* chamber, at 22 ºC.

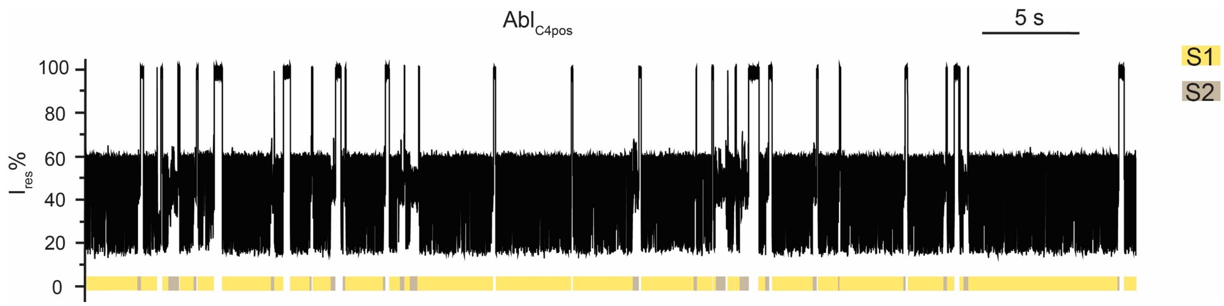


**Supplementary Fig. 6.** A representative trace of Abl$_{C4pos}$ trapped in a ClyA-AS nanopore. Signal pattern S1 (yellow) and S2 (brown) are observed when Abl$_{C4pos}$ is trapped within nanopore. The current traces were collected at −80 mV in 150 mM NaCl, 100 mM Tris-HCl, pH 7.5, with ~100 nM Abl$_{C4pos}$ kinase added to *cis* chamber, at 22 ºC.

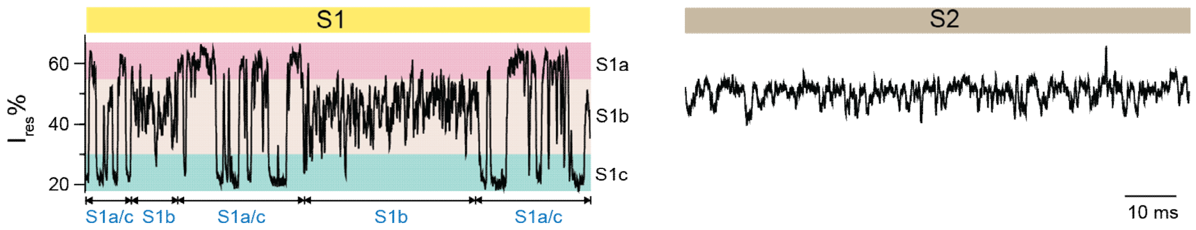


**Supplementary Fig. 7.** Zoom in trace of S1 and S2 states from apo Abl trapping signal. Three sub-states were observed within S1, namely, S1a, S1b, and S1c. The state transition pathways were: S1↔S2, S1a/c↔S1b, and S1a↔S1c. The current traces were collected at −80 mV in the buffer 150 mM NaCl, 100 mM Tris-HCl, pH 7.5, with ~100 nM Abl kinase added to *cis* chamber, at 22 ºC.

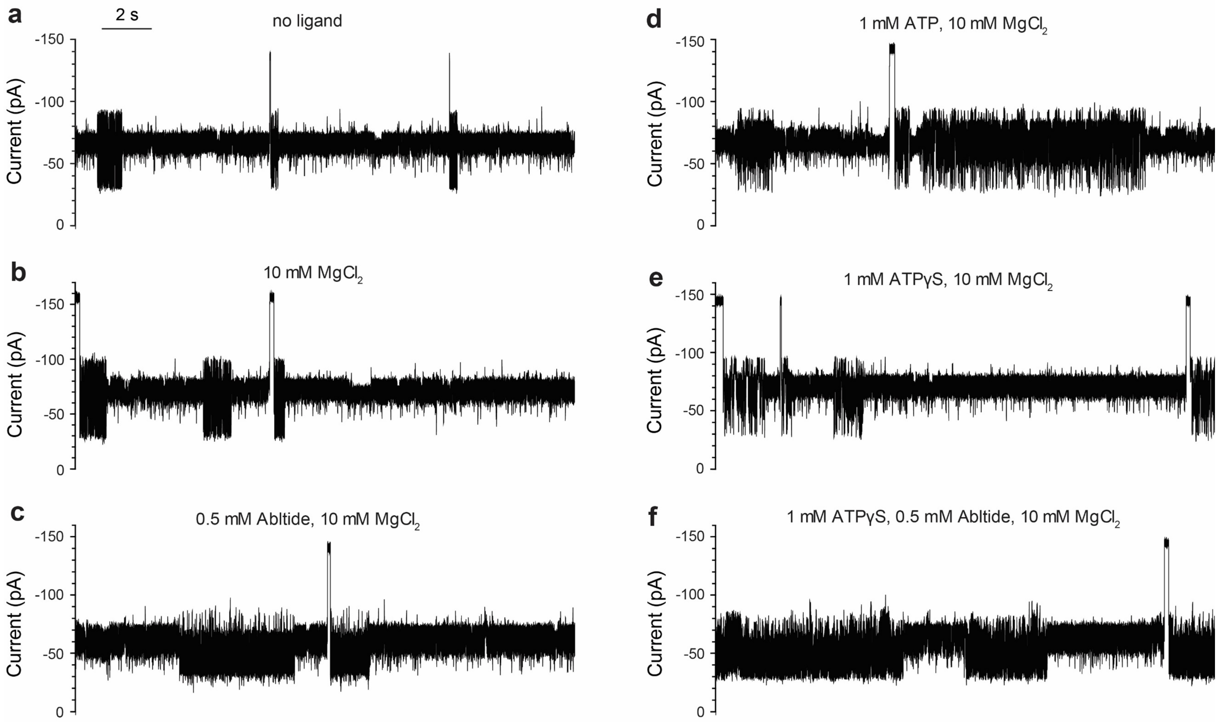


**Supplementary Fig. 8.** Representative traces of ligand interactions with $_{N4pos}$Abl. (a) Apo $_{N4pos}$Abl, (b-f) $_{N4pos}$Abl in the presence of 10 mM $MgCl_2$ (b), 0.5 mM Abltide and 10 mM $MgCl_2$ (c), 1 mM ATP and 10 mM $MgCl_2$ (d), 1 mM ATPγS and 10 mM $MgCl_2$ (e), 0.5 mM Abltide, 1 mM ATPγS and 10 mM $MgCl_2$ (f). Amino acid sequence of Abltide: KKGEAIYAAPFA. The current traces were collected at −80 mV in the buffer 150 mM NaCl, 100 mM Tris-HCl, pH 7.5, at 22 ºC, with ~100 nM $_{N4pos}$Abl and ligands added to *cis*.

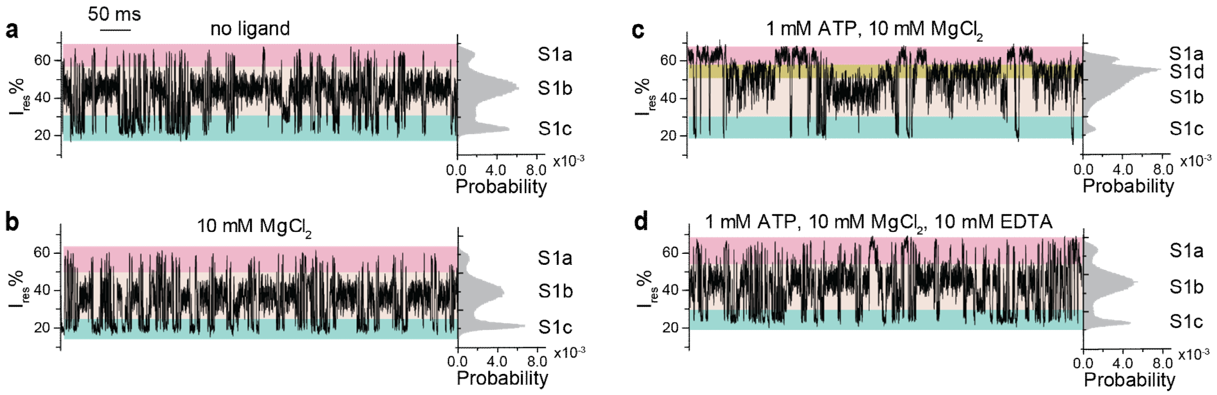


**Supplementary Fig. 9.** Effect of $MgCl_2$ on ATP binding with $_{N4pos}$Abl. (a-d) Representative traces and corresponding histograms of S1 of apo $_{N4pos}$Abl (a) and $_{N4pos}$Abl in the presence of 10 mM $MgCl_2$ (b), 1 mM ATP and 10 mM $MgCl_2$ (c), 1 mM ATP, 10 mM $MgCl_2$ and 10 mM EDTA (d). The current traces were collected at −80 mV in the buffer 150 mM NaCl, 100 mM Tris-HCl, pH 7.5, at 22 ºC, with ~100 nM $_{N4pos}$Abl and ligands added to *cis*.

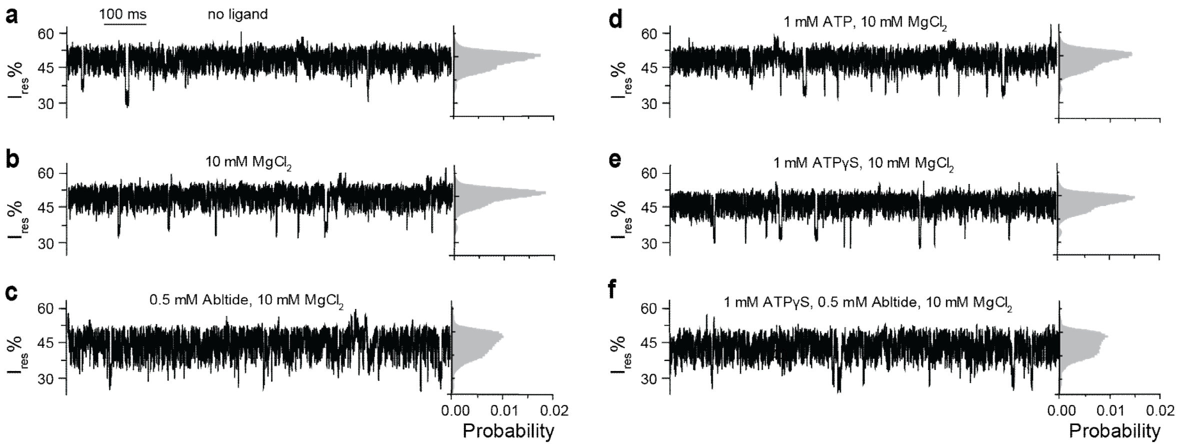


**Supplementary Fig. 10.** Representative traces and corresponding histograms of S2 state during ligand interactions with $_{N4pos}$Abl. (a-f) Representative traces and corresponding histograms of S2 of apo $_{N4pos}$Abl (a) and $_{N4pos}$Abl in the presence of 10 mM $MgCl_2$ (b), 0.5 mM Abltide and 10 mM $MgCl_2$ (c), 1 mM ATP and 10 mM $MgCl_2$ (d), 1 mM ATPγS and 10 mM $MgCl_2$ (e), 0.5 mM Abltide, 1 mM ATPγS and 10 mM $MgCl_2$ (f). Amino acid sequence of Abltide: KKGEAIYAAPFA. The current traces were collected at −80 mV in the buffer 150 mM NaCl, 100 mM Tris-HCl, pH 7.5, at 22 ºC, with ~100 nM $_{N4pos}$Abl and ligands added to *cis*.

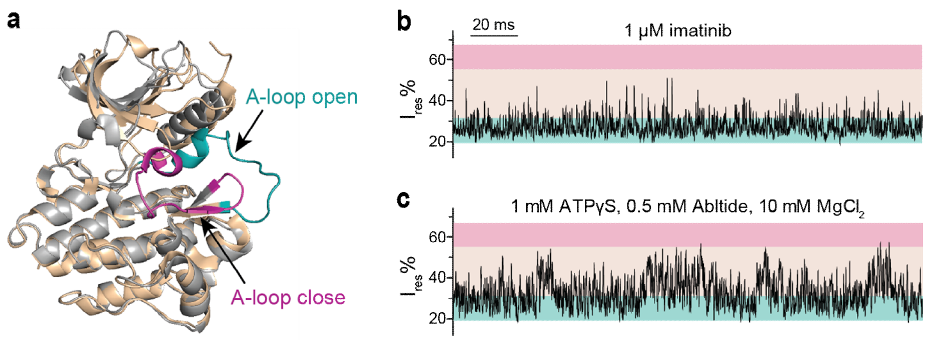


**Supplementary Fig. 11.** Comparison of $_{\text{N4pos}}$Abl binding with imatinib and $_{\text{N4pos}}$Abl binding with ATPγS and Abltide. (a) Superposition of imatinib bound Abl kinase (PDB: 2HYY, Abl in grey, A-loop in magenta) with ATP-peptide bound Abl kinase structures (PDB: 2G1T, Abl in wheat, A-loop in teal). (b-c) Representative traces of $_{\text{N4pos}}$Abl with 1 μM imatinib (b) and 1 mM ATPγS, 0.5 mM Abltide and 10 mM $MgCl_2$ (c). The current traces were collected at −80 mV in the buffer 150 mM NaCl, 100 mM Tris-HCl, pH 7.5, at 22 ºC, with ~100 nM $_{\text{N4pos}}$Abl and ligands added to *cis*.

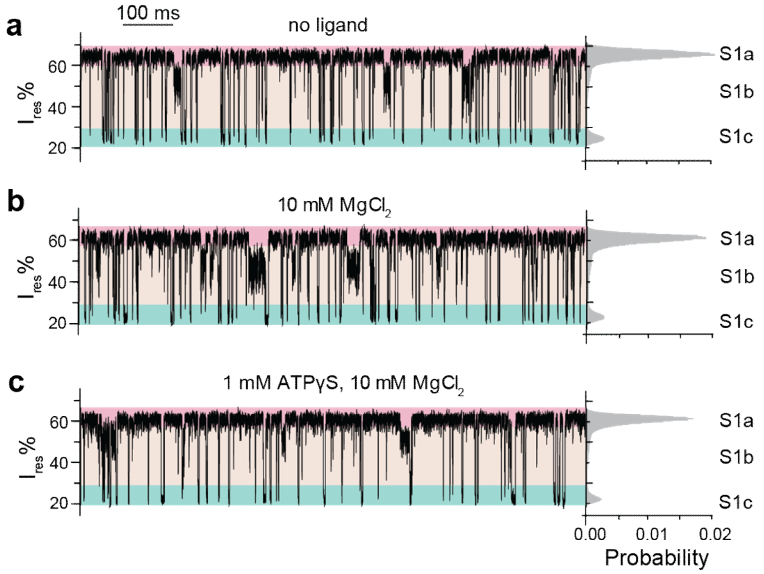


**Supplementary Fig. 12.** Interaction of ATPγS with $_{N4pos}$G321V. (a-c) Representative traces and corresponding histograms of S1 of apo $_{N4pos}$G321V (a), $_{N4pos}$G321V in the presence of 10 mM $MgCl_2$ (b), and $_{N4pos}$G321V in the presence of 1 mM ATPγS, 10 mM $MgCl_2$. The current traces were collected at −80 mV in the buffer 150 mM NaCl, 100 mM Tris-HCl, pH 7.5, at 22 ºC, with ~100 nM $_{N4pos}$G321V and ATPγS and $MgCl_2$ added to *cis*.

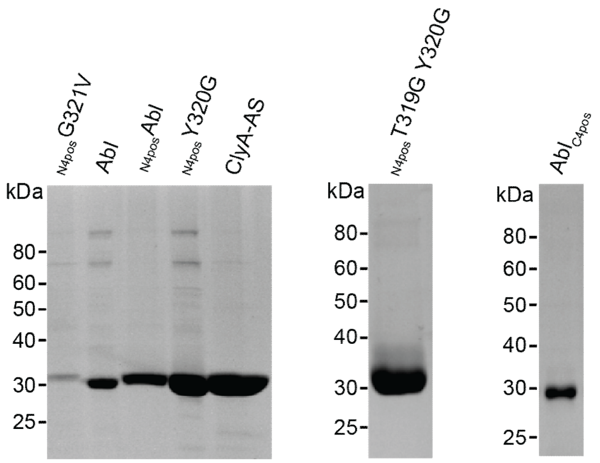


**Supplementary Fig. 13.** SDS-PAGE analysis (12% SDS gels) of ClyA-AS and Abl kinases (Abl, $_{N4pos}$Abl, Abl$_{C4pos}$, $_{N4pos}$G321V, $_{N4pos}$Y320G, and $_{N4pos}$T319G Y320G). Three batches of ClyA-AS protein were purified independently. Two batches of Abl, $_{N4pos}$Abl and $_{N4pos}$G321V proteins were purified independently. Once batch of $_{N4pos}$Y320G, $_{N4pos}$T319G Y320G and Abl$_{C4pos}$ were purified.

**Supplementary Table 1**. Michaelis-Menten kinetics analysis of Abl kinases.

| Abl kinase | $K_m$ (μM) | $V_{max}$ (μM/min) | $k_{cat}$ ($min^{-1}$) | $k_{cat}/K_m$ ($min^{-1}μM^{-1}$) |
|---|---|---|---|---|
| Abl | 167.6 ± 47.7 | 12.8 ± 1.3 | 426.7 ± 43.0 | 2.5 ± 0.9 |
| $Abl_{C4pos}$ | 64.1 ± 18.9 | 9.68 ± 0.71 | 322.7 ± 23.7 | 5.0 ± 1.3 |
| $_{N4pos}Abl$ | 194.2 ± 40.2 | 13.3 ± 1.1 | 443.7± 35.1 | 2.3 ± 0.9 |
| $_{N4pos}Y320G$ | 468.2 ± 95.6 | 20.3 ± 2.1 | 676.7 ± 68.8 | 1.4 ± 0.7 |
| $_{N4pos}T319G$ Y320G | 382.9 ± 106.0 | 29.8 ± 3.9 | 993.3 ± 130.0 | 2.6 ± 1.2 |
| $_{N4pos}G321V$ | ND | ND | ND | ND |

Data are presented as mean ± SD, n=3 independent replicates. Source data are provided as a Source Data file. Abbreviation: ND, not determined.

**Supplementary Table 2**. Voltage dependent trapping time analysis of Abl kinases.

| Abl kinase | Voltage (mV) | $\tau_{trapping}$ (s), Mean ± S.D. | Pore number | Total event number |
|---|---|---|---|---|
| Abl | 80 | 0.058 ± 0.026 | 5 | 1068 |
| | 90 | 0.317 ± 0.156 | 6 | 718 |
| | 100 | 2.330 ± 0.662 | 6 | 661 |
| | 110 | 2.116 ± 0.621 | 3 | 379 |
| | 120 | 1.964 ± 0.081 | 2 | 173 |
| $_{N4pos}$Abl | 60 | 0.136 ± 0.020 | 3 | 437 |
| | 70 | 4.928 ± 1.531 | 3 | 522 |
| | 80 | 21.478 ± 3.599 | 5 | 753 |
| | 90 | 6.859 ± 1.360 | 3 | 349 |
| | 100 | 6.544 ± 1.920 | 3 | 181 |
| $Abl_{C4pos}$ | 60 | 0.288 ± 0.064 | 3 | 631 |
| | 70 | 0.986 ± 0.217 | 3 | 528 |
| | 80 | 1.379 ± 0.141 | 3 | 657 |
| | 90 | 0.822 ± 0.164 | 3 | 1066 |
| | 100 | 0.647 ± 0.017 | 3 | 821 |

Source data are provided as a Source Data file.

**Supplementary Table 3.** Characteristics of apo $_{N4pos}$Abl and $_{N4pos}$G321V current signals at -80 mV.

| State | State population % (P) | | Residual Current ($I_{res}$%) | | State Dwell time (τ) | |
|---|---|---|---|---|---|---|
| | $_{N4pos}$Abl | $_{N4pos}$G321V | $_{N4pos}$Abl | $_{N4pos}$G321V | $_{N4pos}$Abl | $_{N4pos}$G321V |
| S1 | 4.6 ± 1.5 | 3.1* | 19~69 | 21~69 | 0.55 ± 0.07 s | 0.79 s* |
| S2 | 95.4 ± 1.5 | 96.9* | 38~58 | 40~59 | 12.25 ± 3.16 s | 24.46 s* |
| S1a | 0.8 ± 0.1 | 2.5 ± 0.1 | 58~69 | 60~69 | 1.1 ± 0.1 ms | 9.1 ± 0.4 ms |
| S1b | 2.8 ± 0.9 | 0.2 ± 0.0 | 31~58 | 30~60 | 10.2 ± 0.2 ms | 6.0 ± 0.4 ms |
| S1c | 1.0 ± 0.1 | 0.4 ± 0.1 | 19~31 | 21~30 | 1.3 ± 0.2 ms | 1.6 ± 0.3 ms |
| S1a/c | 1.8 ± 0.1 | 3.0 ± 0.0 | 19~69 | 21~69 | 6.5 ± 0.4 ms | 99.9 ± 6.2 ms |

Data are presented as mean ± SD, n=3 independent replicates. Source data are provided as a Source Data file. Note, for $_{N4pos}$G321V, S1 (n=231) and S2 (n=222) events were combined from 16 experiments and used for $\tau_{S1}$, $\tau_{S2}$ fitting, respectively. $P_{S1}$ and $P_{S2}$ were calculated by $\tau_{S1}$ and $\tau_{S2}$ described in Methods section. The asterisk (*) indicates that the data was fitted after combining events from different experiments.

**Supplementary Table 4.** Occupancy of S1 sub-states of $_{N4pos}$Abl under different conditions.

| State occupancy (%) | S1a/S1 | S1b/S1 | S1c/S1 | S1d/S1 |
|---|---|---|---|---|
| No ligand | 18.6 ± 0.0 | 59.1 ± 0.1 | 22.3 ± 0.1 | 0 |
| 10mM $MgCl_2$ | 13.3 ± 2.6 | 62.1 ± 2.9 | 25.0 ± 3.2 | 0 |
| 1mM ATP, 10mM $MgCl_2$ | 19.3 ± 2.2 | 36.3 ± 3.0 | 4.5 ± 0.1 | 39.8 ± 2.1 |
| 1mM ATPγS, 10mM $MgCl_2$ | 30.0 ± 2.0 | 42.4 ± 3.5 | 3.4 ± 1.1 | 24.1 ± 1.6 |
| 0.5mM Abltide, 10mM $MgCl_2$ | 2.4 ± 0.3 | 93.6 ± 0.3 | 4.0 ± 0.6 | 0 |
| 1mM ATPγS, 0.5mM Abltide 10mM $MgCl_2$ | 4.1 ± 0.5 | 58.0 ± 2.0 | 37.9 ± 2.5 | 0 |

Data are presented as mean ± SD, n=3 independent replicates. Source data are provided as a Source Data file.

**Supplementary Table 5.** Trapping time of different Abl constructs and their S1, S2 dwell times at -80 mV.

| Abl kinase | $\tau_{trapping}$ (s) | $\tau_{S1}$ (s) | $\tau_{S2}$ (s) |
|---|---|---|---|
| $_{N4pos}$Abl | 21.48 ± 3.60 | 0.55 ± 0.07 | 12.25 ± 3.16 |
| $_{N4pos}$Y320G | 16.00 ± 2.46 | 0.45 ± 0.06 | 5.94 ± 0.41 |
| $_{N4pos}$T319G Y320G | 6.71 ± 1.64 | 0.13 ± 0.05 | 3.23 ± 1.38 |
| $_{N4pos}$G321V | 23.80 ± 4.66 | 0.79* | 24.46* |

Data are presented as mean ± SD, n⩾3 independent replicates. Source data are provided as a Source Data file. Note, for $_{N4pos}$G321V, we were not able to collect enough events to derive the $\tau_{S1}$ and $\tau_{S2}$ from each single pore due to the slow S1/S2 transitions. Therefore, S1 (n=231) and S2 (n=222) events from 16 independent pores were combined to calculate the average duration. The asterisk (*) indicates that the data was fitted after combining events from different experiments.

**Supplementary Table 6.** Kinetic and thermodynamic parameters for apo $_{N4pos}$Abl.

| State transition rate constant | | Free energy difference between states | |
|---|---|---|---|
| $k_{S1\rightarrow S2}$, $s^{-1}$<br>$k_{S2\rightarrow S1}$, $s^{-1}$ | 1.86 ±0.24<br>0.09 ± 0.02 | $\Delta G_{S1, S2}$, $KJ*mol^{-1}$ | 7.57±0.91 |
| $k_{S1a/c\rightarrow S1b}$, $s^{-1}$<br>$k_{S1b\rightarrow S1a/c}$, $s^{-1}$ | 154.1 ± 9.6<br>97.9 ± 1.5 | $\Delta G_{S1a/c, S1b}$, $KJ*mol^{-1}$ | 1.11 ± 0.17 |
| $k_{S1a\rightarrow S1c}$, $s^{-1}$<br>$k_{S1c\rightarrow S1a}$, $s^{-1}$ | 974.0 ± 142.0<br>824.0 ± 141.5 | $\Delta G_{S1a, S1c}$, $KJ*mol^{-1}$ | 0.42 ± 0.21 |

Data are presented as mean ± SD, n=3 independent replicates. Source data are provided as a Source Data file.

**Supplementary Table 7.** Primers for Abl variants cloning.

| Application | Primer name | Sequence (5’ to 3’) |
|---|---|---|
| Insert KRKKSGG to the N-terminus of Abl WT, generating $_{N4pos}$Abl | N4pos_F | AAACGTAAGAAAAGCGGAGGTTCCCCCAACTACGACAAGTGGGAG |
| | N4pos_R | ACCTCCGCTTTTCTTACGTTTTGCATTGGATTGGAAGTACAGG |
| Insert GGSKKRK to the C-terminus of Abl WT, generating Abl$_{C4pos}$ | C4pos_F | GGAGGTAGCAAGAAACGCAAATAATAACATTGGAAGTGGATAACG |
| | C4pos_R | TTTGCGTTTCTTGCTACCTCCTTTCCCCAGCTCCTTTTCCACTTC |
| Insert Y320G to $_{N4pos}$Abl, generating $_{N4pos}$Y320G | Y320G_F | GTTCATGACCGGAGGGAACCTCCTGGACTAC |
| | Y320G_R | GAGGTTCCCTCCGGTCATGAACTCAGTGATGAT |
| Insert G321V to $_{N4pos}$Abl, generating $_{N4pos}$G321V | G321V_F | CATGACCTACGTGAACCTCCTGGACTACC |
| | G321V_R | GTTCACGTAGGTCATGAACTCAGTGATGATATAG |
| Insert T319G Y320G to $_{N4pos}$Abl, generating $_{N4pos}$T319G Y320G | T319G Y320G_F | GAGTTCATGGGCGGAGGGAACCTCCTGGAC |
| | T319G Y320G_R | TCCGCCCATGAACTCAGTGATGATATAGAAC |
